# Effectiveness of Melodic Intonation Therapy in Chinese Mandarin on Non-fluent Aphasia in Patients After Stroke: A Randomized Control Trial

**DOI:** 10.3389/fnins.2021.648724

**Published:** 2021-07-23

**Authors:** Xiao-Ying Zhang, Wei-Yong Yu, Wen-Jia Teng, Meng-Yang Lu, Xiao-Li Wu, Yu-Qi Yang, Chen Chen, Li-Xu Liu, Song-Huai Liu, Jian-Jun Li

**Affiliations:** ^1^School of Rehabilitation Medicine, Capital Medical University, Beijing, China; ^2^China Rehabilitation Science Institute, Beijing, China; ^3^Beijing Key Laboratory of Neural Injury and Rehabilitation, Beijing, China; ^4^Center of Neural Injury and Repair, Beijing Institute for Brain Disorders, Beijing, China; ^5^Music Therapy Center, Department of Psychology, China Rehabilitation Research Center, Beijing, China; ^6^Department of Imaging, China Rehabilitation Research Center, Beijing, China; ^7^Department of Neurorehabilitation, China Rehabilitation Research Center, Beijing, China; ^8^Department of Music Education, Xinghai Conservatory of Music, Guangzhou, China

**Keywords:** stroke, non-fluent aphasia, melodic intonation therapy, Chinese Mandarin, music therapy

## Abstract

Melodic intonation therapy (MIT) positively impacts the speech function of patients suffering from aphasia and strokes. Fixed-pitch melodies and phrases formulated in MIT provide the key to the target language to open the language pathway. This randomized controlled trial compared the effects of music therapy-based MIT and speech therapy on patients with non-fluent aphasia. The former is more effective in the recovery of language function in patients with aphasia. Forty-two participants were enrolled in the study, and 40 patients were registered. The participants were randomly assigned to two groups: the intervention group (*n* = 20; 16 males, 4 females; 52.90 ± 9.08 years), which received MIT, and the control group (*n* = 20; 15 males, 5 females; 54.05 ± 10.81 years), which received speech therapy. The intervention group received MIT treatment for 30 min/day, five times a week for 8 weeks, and the control group received identical sessions of speech therapy for 30 min/day, five times a week for 8 weeks. Each participant of the group was assessed by a Boston Diagnostic Aphasia Examination (BDAE) at the baseline (t1, before the start of the experiment), and after 8 weeks (t2, the experiment was finished). The Hamilton Anxiety Scale (HAMA) and Hamilton Depression Scale (HAMD) were also measured on the time points. The best medical care of the two groups is the same. Two-way ANOVA analysis of variance was used only for data detection. In the spontaneous speech (information), the listening comprehension (right or wrong, word recognition, and sequential order) and repetitions of the intervention group were significantly higher than the control group in terms of the cumulative effect of time and the difference between groups after 8 weeks. The intervention group has a significant time effect in fluency, but the results after 8 weeks were not significantly different from those in the control group. In terms of naming, the intervention group was much better than the control group in spontaneous naming. Regarding object naming, reaction naming, and sentence completing, the intervention group showed a strong time accumulation effect. Still, the results after 8 weeks were not significantly different from those in the control group. These results indicate that, compared with speech therapy, MIT based on music therapy is a more effective musical activity and is effective and valuable for the recovery of speech function in patients with non-fluent aphasia. As a more professional non-traumatic treatment method, MIT conducted by qualified music therapists requires deeper cooperation between doctors and music therapists to improve rehabilitating patients with aphasia. The Ethics Committee of the China Rehabilitation Research Center approved this study (Approval No. 2020-013-1 on April 1, 2020) and was registered with the Chinese Clinical Trial Registry (Registration number: Clinical Trials ChiCTR2000037871) on September 3, 2020.

## Introduction

Stroke constitutes one of the leading causes of long-term disability worldwide (Benjamin et al., 2017). Of the major neurological deficits, language function disorder is the main symptoms for stroke-related impairments, which are defined as aphasia in the clinic. Aphasia is a kind of acquired loss or impairment of the ability to communicate by language following brain damage, which is usually in the left hemisphere (Wade et al., 1986). Aphasia is part of the most common complications that occurred in one-third after stroke, about 21–38% of the patients are correlated with different degrees of symptoms in stroke survivors (Dickey et al., 2010). Aphasia is often subdivided into fluent and non-fluent aphasia. Non-fluent aphasia generally results from a stroke in the left frontotemporal regions and is characterized by slow, effortful speech (Meulen et al., 2016). It mainly presents an oral expression barrier, with relatively good comprehension, and difficulty in understanding grammatical words, order words, sentences, retelling, naming, reading, and writing in varying degrees (Fazio et al., 2009). Non-fluent aphasia mainly includes Broca’s aphasia, complete aphasia, and so on.

Melodic intonation therapy (MIT) is a formalized impairment-based approach of language rehabilitation that uses melodic and rhythmic elements of intoning phrases and words to assist in speech recovery in patients with Broca’s aphasia (Albert et al., 1973). MIT was developed by a group of neurologic researchers in the early 1970s and, now, is a hierarchically structured treatment program identified by the American Academy of Neurology as an effective form of output-focused language therapy (Helm-Estabrooks and Albert, 1991; Assessment, 1994). The basic rationale for MIT emphasizes the use of rhythmic musical elements to engage language-capable regions of the undamaged right hemisphere (Helm-Estabrooks and Albert, 2004). Considering the dominant role of music processing in the right hemisphere, MIT uses the comprehensive characteristics of music, rhythm, and speech output, and uses the proprioceptive input of the left hand to participate in the control of the sensory motor network and oral output (Schuppert et al., 2000; Gentilucci and Dalla Volta, 2008; Norton et al., 2009). Among the musical parts in MIT, the intoned-speech technique is a musical stylization of the normal speech prosody using a few pitches, usually only two or four, separated by a third or a fourth, and a simple rhythm, quarter or eighth notes (Sparks, 2008), formulated into a short melody to represent the trained phrases on a slow tempo.

MIT was originally applied in the English-speaking patients (Norton et al., 2009). In recent years, there are several literatures that reported that non-English MIT were applied in clinical aphasia populations (Cortese et al., 2015; Tabei et al., 2016), including non-English linguistic patients such as Italian (Cortese et al., 2015), Japanese (Tabei et al., 2016), Romanian (Popovici, 1995), Persian (Bonakdarpour et al., 2003), French (Zumbansen et al., 2014), Dutch (Van der Lugt-van Wiechen and Verschoor, 1987), and Caucasian (Breier et al., 2010) with comparable clinical results. However, most of these studies were case studies, minimal sample studies, meta-analysis, and mechanism researches in chronic aphasia (Van der Meulen et al., 2012), and no large sample studies focused on East Asian languages, especially for Chinese Mandarin. The literature has shown now that MIT is more effective than normal speech therapy in different language chronic aphasia. A group study of 11 chronic non-fluent English-speaking aphasic patients examined by Wan et al. (2014) reported an improved communicative effectiveness and verbal fluency after MIT, and associated with structural changes in the white matter underlying the right inferior frontal gyrus. In Italian and French-speaking patients (Zumbansen et al., 2014; Cortese et al., 2015), the MIT group showed a significant improvement after 16 weeks and also has the same effect in spontaneous speech at the 6-months follow-up. Japanese is the best close to Chinese Mandarin in Eastern Asian language family. In Japanese MIT, Tabei et al. (2016) reported a marked improvement in following an intensive 9-day training on one Japanese MIT. Following MIT-J, the arcuate fasciculus of a part of the right hemisphere was improved by increased neural processing efficiency. Chen et al. (2020) reported that 17 patients with non-fluent aphasia in Chinese Mandarin had a significant improvement in the score of Western Aphasia Battery (WAB) scale after MIT treatment. Therefore, it can be seen that, although MIT is effective in clinical interventions in East Asian languages, especially for Mandarin Chinese (MIT-C), a large sample evidence is still needed.

It is reported that the number of new stroke cases that occurred in China was about 2.6–4.7 million in 2019 (Wang et al., 2019), ranking the first in the world (Johnson et al., 2019). Therefore, the MIT in Chinese for patients with aphasia after stroke is particularly necessary. Unlike the multisyllable pronunciation in the Western language, Chinese mandarin is monosyllabic pronunciation, and also, one syllable has four tones, each of which represents a different meaning. According to the regularity of melody and rhythm of speech, syllabic pitch is a relative-pitch system using musical notes and a series of “peculiar symbols” that would represent the relative pitch and relative duration of each spoken syllable of an utterance (Chow and Brown, 2018). In this basic terms of pronunciation rule, MIT is more suitable with short melodies in Chinese character of word and sound. Zhang et al. reported in a case study that according to the three levels of language rehabilitation, the content of Chinese MIT can be divided into three steps (Zhang et al., 2016): (1) 1–3-words sentence, (2) 4–6-word sentence, (3) 7-word sentence and 7 above (Zhang et al., 2016). This randomized controlled trial (RCT) is to observe the behavioral efficacy of existing treatment paradigm of MIT-C in clinical intervention for Chinese aphasia. We used the RCT design to compare the therapeutic effects of MIT-C and speech therapy in patients with non-fluent aphasia whose mother tongue is Chinese, and to explore the specific target curative effect of the existing MIT-C clinical operation paradigm with aphasia.

## Subjects and Methods

This study was approved by the Ethics Committee of China Rehabilitation Research Center (CRRC) (approval No. 2020-013-1) on April 1, 2020 (Supplementary File 1), and informed consent (Supplementary File 2) was obtained from the participants, relatives, or guardians before commencing the study. The study trial was registered with the Chinese Clinical Trial Registry (Registration No. ChiCTR2000037871) on September 3, 2020.

### Participants

Forty participants were recruited from CRRC, Beijing. The inclusion criteria were as follows: (1) diagnosed with fMRI or CT imaging, showing left ischemic stroke or hemorrhagic stroke; (2) The ninth language score on the National Institutes of Health Stroke Scale (NIHSS) (Farooque et al., 2020) is 1—mild to moderate aphasia and 2—severe aphasia. (3) meeting the diagnostic criteria for non-fluent aphasia: less active speech expression, lack of fluency in speaking, acceptable hearing ability, can give a sign of yes/no questions, willing to express, good cooperation, and emotional stability (Wang et al., 2019); (4) Aphasia for more than 15 days after stroke, hospitalized patients; (4) aged 18–70; (5) tolerance to lying therapy for more than half an hour without postural hypotension; (6) The medication and other brain metabolism enhancers are the same; physical therapy, occupational therapy, and routine care are the same. (7) None of the participants had professional musical experience. (8) Patients and their families provided written informed consent to participate in this study. The exclusion criteria were (1) severe auditory dysfunction; (2) having epilepsy, malignant arrhythmia, or other serious physical diseases; and (3) patients with mental symptoms and obvious emotional agitation. Criteria for withdrawal and termination: patients could be terminated if their condition changed, if they were discharged from the hospital, or if they voluntarily withdrew. Forty participants completed the experiment. Two participants were withdrawn from the study because they did not meet the inclusion criteria. The data of participants’ characteristics are shown in Table 1.

**TABLE 1:** Participants’ characteristics in this study.

	Intervention group	Control group	*t*	*p*
**Total Number**	20	20		
**Gender**				
Male	16	15		
Female	4	5		
**Age**	52.90 ± 9.08	54.05 ± 10.81	0.5089	0.5845
months since injury	2.57 ± 1.74	1.96 ± 1.38	0.2677	0.865
**Stoke classification**				
Left cortical ischemic	10	14		
Left cortical hemorrhagic	10	6		
**Non-fluent aphasia**				
**classification**				
Global aphasia	9	12		
Broca’s aphasia	8	7		
Transcortical mixing	3	1		

*Data were expressed as a number in total number, gender, years since injury, stoke classification and non-fluent aphasia classification. Other data were expressed as the mean ± SD, and analyzed by paired t-test. Intervention group: melodic intonation therapy group; Control group: speech therapy group. P > 0.05 indicates no significant difference between the two groups.*

### Study Design

The study was a randomized controlled trial with a pre-test–post-test design. It included two groups: the intervention group (*n* = 20) and the control group (*n* = 20). This study adopts a double-blind design—neither the participants nor the data analyst knows which group of data is being tested and analyzed. The intervention group received melodic intonation therapy, while the control group received speech therapy. The study was conducted from April 2020 to October 2020 at CRRC. The costs involved in this trial are all funded by the 2020CZ-10 scientific research project of the Chinese Institute of Rehabilitation Sciences (CIRS). This is a national non-profit foundation plan and has been approved by the Ministry of Finance of China.

### Procedure

**FIGURE 1:**
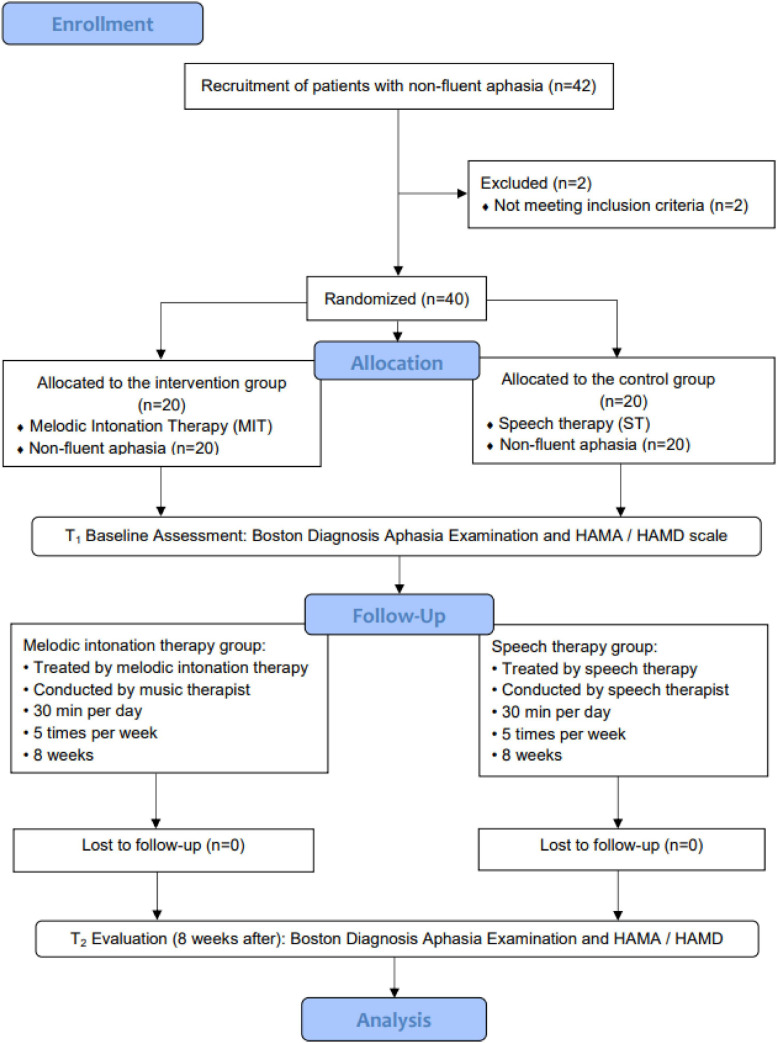
Flow diagram, consort flowchart for participants’ recruitment and allocation.

After obtaining approval from the Scientific Research Foundation of CIRS, participants were screened by the neurorehabilitation specialists. Patients who were diagnosed as non-fluent aphasia in the ninth language score on the NIHSS are 1—mild-to-moderate aphasia and 2—severe aphasia and were referred to the Music Therapy Department at CRRC. Participants were reviewed by the researchers to identify potential interventional objectives based on the inclusion and exclusion criteria of the study. Once potential participants were identified, an invitation inform to the study was sent to their family members. The inform included the purpose, procedures, risks, benefits, confidentiality, and participants’ rights. Once we acquired the consent forms, the participants were assessed by professional evaluators for the modified Boston Diagnostic Aphasia Examination (BDAE) (Fong et al., 2019) to determine non-fluent aphasia types. The clinical researchers screened patients based on BDAE scores to confirm whether they had an abnormal speech function. After the screening, computer-generated sequences (by Excel 2013, Microsoft office software, Seattle, WA, United States) were used to randomly assign the patients into the two groups. The participants in the intervention group were treated by melodic intonation therapy for 8 weeks by registered music therapists, while participants in the control group were treated with speech therapy for 8 weeks. The enrollment and allocation of participants are shown in Figure 1.

Figure 1 illustrates that 42 participants were enrolled in the study, and 2 participants withdrew from the study because of not meeting the inclusion criteria (*n* = 2). The intervention group was treated with melodic intonation therapy (*n* = 20) by a music therapist, while the control group was treated with speech therapy (*n* = 20) by speech therapists. Two times evaluation was conducted during the whole period: t1 (baseline) and t2 (after 8 weeks). The data analysis included a sample of 40 non-fluent aphasia patients.

### Interventions

Once the participants for each group were identified, an intervention delivery schedule was developed. All patients in the intervention group received MIT training. Each patient was trained for 30 min per session, five sessions a week, for 8 weeks. The training process is carried out by music therapy professionals who have been trained in neurological music therapy (NMT) and have obtained a registered music therapist license to ensure the music professionalism of the intervention. The intervention steps of MIT strictly follow the operational steps of Chinese Mandarin MIT (Zhang et al., 2016).

**FIGURE 2:**
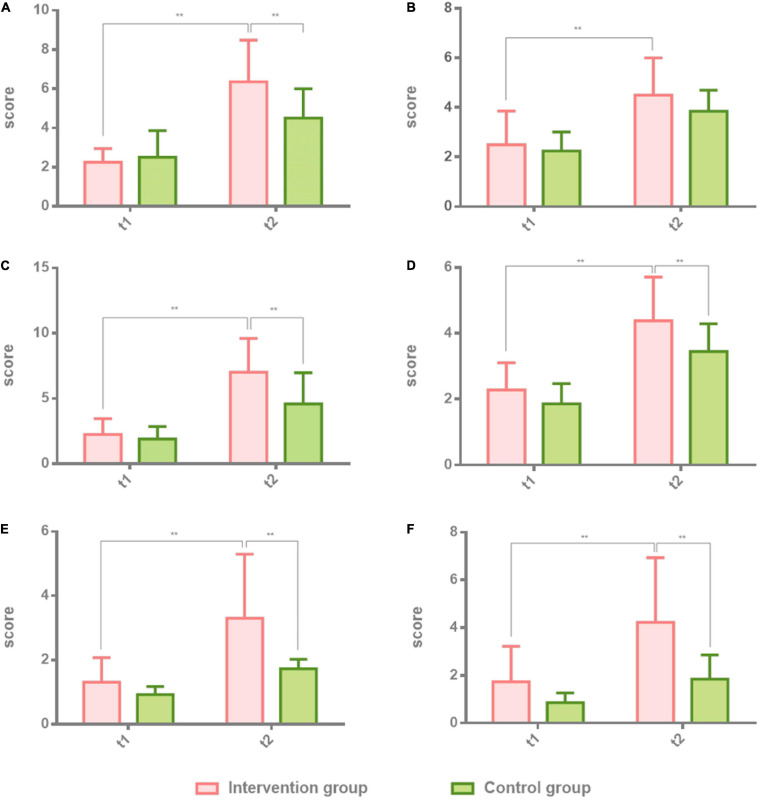
Comparison of spontaneous speech, repetition, and listening comprehension in patients with non-fluent aphasia with the intervention group and the control group. The intervention group: melodic intonation therapy group; the control group: speech therapy group. **(A–F)** Information, fluency, repetition, true or false, word recognition, and sequential commands. Data are expressed as mean ± SD (*n* = 20) and analyzed by repeated-measures analysis of variance. *p < 0.05, **p < 0.01. t_1_, baseline; t_2_, after 8 weeks.


According to the different three levels of speech rehabilitation, the music therapist trained the aphasia patients to intone and chant the targeted speech items, like “*sprenchsang*,” (Helm-Estabrooks et al., 2013) and then fade slowly with tapping to let the patients speak out the targeted sentences in the first level. The music therapist leads the patients to sing and speak out in the same way in the second and third levels; the only difference is the length of the melodic target language (the second level is 5–9-word sentences, and the third level is 10-word sentences and above). All the melodic phrases are noted according to the natural phonic pitches of targeted Mandarin sentences; the specific 12-item implementation contents of MIT are shown in Figure 2. The music therapist uses a keyboard or guitar to accompany while they are singing the melody with the patients. According to the different types of damage in non-fluent aphasia, in the intervention group and the control group of this study (Table 1), there are 21 patients with global aphasia (*n* = 9, *n* = 12), 15 patients with Broca’s aphasia (*n* = 8, *n* = 7), and 4 patients with transcortical mixed aphasia (*n* = 3, *n* = 1). According to the MIT training content in the Supplementary Figure 2, within 8 weeks, the training objectives for patients with global aphasia in the interventional group are the first and second levels (Supplementary Figure 2, items 1–6), the training objectives of the patients with Broca’s aphasia in the interventional group are the second and third levels (Supplementary Figures 2, 1–10 items), and the patients with mixed transcortical aphasia in the interventional group are the first to third levels (Supplementary Figure 2, items 1–9). The effective behavioral performance of the intervention is that, when the therapist asks the target question, the patient can speak the target language at a natural speed without the melody and rhythm, and the behavior performance can last for more than 3 weeks without regression. The therapy sessions of the two groups were both 30 min per day, five times a week, for a total of eight consecutive weeks. All patients underwent routine treatment during the study period, including taking medication and other care and support.

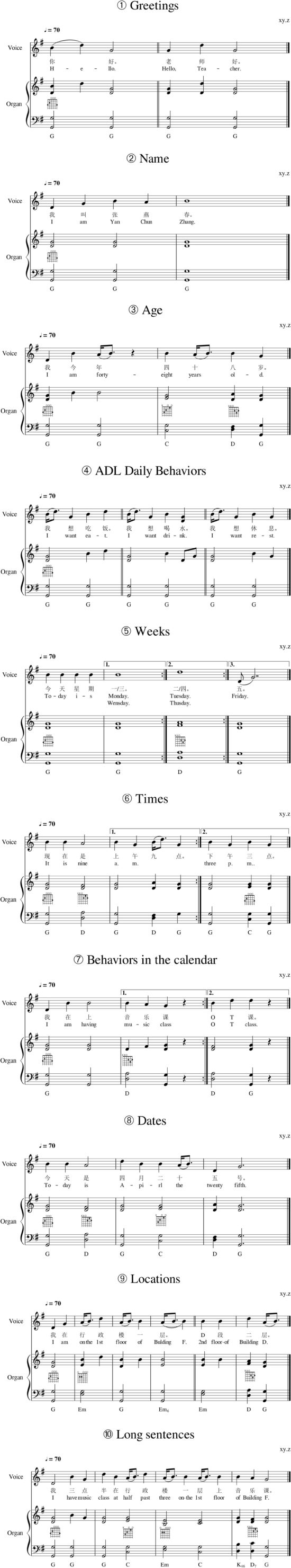


### Measurements

This is to assess the baseline of all the participants in BDAE within 30–40 min before all the therapy started. Then the participants were randomly assigned to the intervention group and the control group. The final evaluation was measured after 8 weeks. The test sessions consisted of the (1) BDAE (Fong et al., 2019) and (2) Hamilton Anxiety Scale (HAMA)/Hamilton Depression Scale (HAMD) (Spreen and Risser, 2003). During the evaluation process, no participants wore a 24-h Holter test or a 24-h ambulatory blood pressure test; no participants had unsealed tracheostomy and had difficulty expecting sputum.

#### Boston Diagnostic Aphasia Examination

BDAE is a measure used in the neuropsychological assessment of aphasia and is currently in its third edition (Fong et al., 2019). It evaluates language skills in aphasia based on perceptual modalities (auditory, visual, and gestural), processing functions (comprehension, analysis, and problem solving), and response modalities (writing, articulation, and manipulation). Administration time ranges from 35 to 45 min. Other tests are sometimes used by neurologists and speech language pathologists on a case-by-case basis, but BDAE is a universal language assessment scale that has been proven, with high reliability and validity, and can be applied to multiple languages. BDAE is a comprehensive, multifactorial battery designed to evaluate a broad range of language impairments that often arise as a consequence of organic brain dysfunction. The examination is designed to go beyond simple functional definitions of aphasia into the components of language dysfunctions (symptoms) that have been shown to underlie the various aphasic syndromes.

BDAE includes four dimensions: spontaneous speech, repetition, listening comprehension, and naming. Among them, spontaneous speech includes information and fluency assessment; listening comprehension includes three assessments of right and wrong questions, word recognition, and sequential instruction; naming includes four items: object naming, spontaneous speech, sentence supplement, and response naming. Finally, the above four subtables are calculated into the total score formula to obtain the aphasia quotient (AQ). Thus, this approach allows for measurement of language-related skills and abilities and neuropsychological analysis from both ideographic and nomothetic bases, as well as a comprehensive approach to the symptom configurations that relate to neuropathologic conditions (Spreen and Risser, 2003).

#### Hamilton Anxiety Scale and Hamilton Depression Scale

The HAMA is a scale commonly used in the clinic to assess the anxiety of patients. The HAMD is the most commonly used scale for clinical evaluation of depression. The HAMD used in this study is a 17-item score battery. Both HAMA and HAMD use a five-level scoring method of 0–4 points. The scores range from asymptomatic to extremely severe. Both of the evaluations are concise and efficient. In this study, HAMA and HAMD have clinical reference values for the positive psychological effect of patients with non-fluent aphasia before and after therapy.

Among those assessments, the BDAE tests and the HAMA/HAMD questionnaires are evaluated by experienced professionals. All the evaluators were registered research assistants who worked as health care professionals with 5 years of clinical experience. The evaluated results were on the consistency test analysis with open-label design.

### Statistical Analysis

The measure data of the two groups were collected at two time points before intervention (t_1_) and 8 weeks later (t_2_). Taking the mean of each group and the standard deviation of the normal distribution, repeated measures of variance (two-way ANOVA) were used to observe intergroup differences, time effects, and intergroup time interaction differences. SPSS statistical software, Version 22.0 (IBM Lenovo, BJ) was used for statistical analysis. The data of 40 patients with non-fluent aphasia who completed this study were analyzed by SPSS 22.0. All the data of the intervention group and the control group were collected before (t1) and after the intervention (t2). Before analysis, basic frequencies were run on the data to screen for missing values and outliers and to establish data entry accuracy. Data were analyzed using a repeated-measures ANOVA to determine the specific effects of the interventions.

## Results

### Effectiveness of the Boston Diagnostic Aphasia Examination Test Results in Patients With Non-fluent Aphasia: The Part of Spontaneous Speech, Repetition, and Listening Comprehension

The first part of the BDAE scale, spontaneous speech, repetition, and listening comprehension, was tested in both groups before the first session and after the last session. Two-way ANOVA was used to analyze the results of the intervention group and the control group at t1 and t2. Individual results were normalized by ruling out the difference of greater dispersion. The effect of the intervention group is higher than the control group. There were significant differences in the intervention group at t2 (8 weeks after) on information (*t*_2_ = 6.35 ± 2.13, *t* = 0.5775, *p* = 0.0002), fluency (*t*_2_ = 4.50 ± 1.50, *t* = 3.975, *p* = 0.0019), which belongs to spontaneous speech, and repetition (*t*_2_ = 7.01 ± 2.61, *t* = 3.975, *p* = 0.0019) in comparison with the control group. There were also significant differences in the intervention group at t2 (8 weeks after) on true or false (*t*_2_ = 4.38 ± 1.33, *t* = 3.134, *p* = 0.0019), word recognition (*t*_2_ = 3.30 ± 2.00, *t* = 0.13, *p* = 0.0001), and sequential commands (*t*_2_ = 4.23 ± 2.70, *t* = 4.591, *p* = 0.0001) in comparison with the control group. Table 2 shows the results of the two groups. Figure 2 shows the comparison results of the two groups.

**TABLE 2:** The results of Boston Diagnosis Aphasia Examination (BDAE) in patients with non-fluent aphasia across the study period for the intervention group and the control group.

			Intervention group (*n* = 20)	Control group (*n* = 20)	*t*	*p*
			Mean ± SD	Mean ± SD		
Spontaneous speech	Information	t_1_	2.25 ± 0.70	4.40 ± 0.86	4.967	0.0001** a
		t_2_	6.35 ± 2.13	6.60 ± 1.32	0.5775	0.0002** b
	Fluency	t_1_	2.50 ± 1.36	2.25 ± 0.77	0.6795	0.0878
		t2	4.50 ± 1.50	3.85 ± 0.85	1.767	0.0001** b
Repetition		t1	2.25 ± 1.22	1.90 ± 0.95	0.5655	0.0001** a
		t_2_	7.01 ± 2.61	4.59 ± 2.39	3.975	0.0019** b
Listening comprehension	True or False	t_1_	2.28 ± 0.83	1.86 ± 0.61	1.415	0.0001** a
		t_2_	4.38 ± 1.33	3.45 ± 0.84	3.134	0.0019** b
	Words recognition	t_1_	1.31 ± 0.77	0.93 ± 0.24	1.106	0.0001** a
		t_2_	3.30 ± 2.00	1.73 ± 0.30	4.583	0.0001** b
	Sequential commands	t_1_	1.74 ± 1.49	0.85 ± 0.41	1.688	0.0001** a
		t_2_	4.23 ± 2.70	1.85 ± 1.02	4.591	0.0001** b
Naming	Objective naming	t_1_	0.66 ± 0.36	1.27 ± 0.48	1.89	0.3064
		t_2_	2.36 ± 1.72	2.22 ± 0.92	0.4337	0.0001** b
	Spontaneous naming	t_1_	0.16 ± 0.11	0.12 ± 0.75	0.4707	0.0042** a
		t_2_	0.50 ± 0.44	0.22 ± 0.11	3.698	0.0001** b
	Sentences completing	t_1_	0.16 ± 0.12	0.19 ± 0.05	0.4052	0.8865
		t_2_	0.50 ± 0.43	0.46 ± 0.14	0.6079	0.0001** b
	Reaction naming	t_1_	0.14 ± 0.11	0.15 ± 0.05	0.0598	0.087
		t_2_	0.62 ± 0.50	0.41 ± 0.12	2.512	0.0001** b
Aphasia Quation (AQ)		t_1_	26.87 ± 9.65	27.85 ± 4.02	0.236	0.0088** a
		t_2_	67.47 ± 22.99	50.71 ± 7.22	4.036	0.0001** b

Intervention group: melodic intonation therapy group; Control group: speech therapy group. Data were expressed as mean ± SD (n = 20), and analyzed by repeated measures analysis of variance. **P < 0.01, remarkable significance; *p < 0.05, significance. Superscript a represents difference factor at the same time between groups, and superscript b represents difference effect of time factor in inter-group.

### Effectiveness of the Boston Diagnostic Aphasia Examination in Patients With Non-fluent Aphasia: Naming and Aphasia Quotient

**FIGURE 3:**
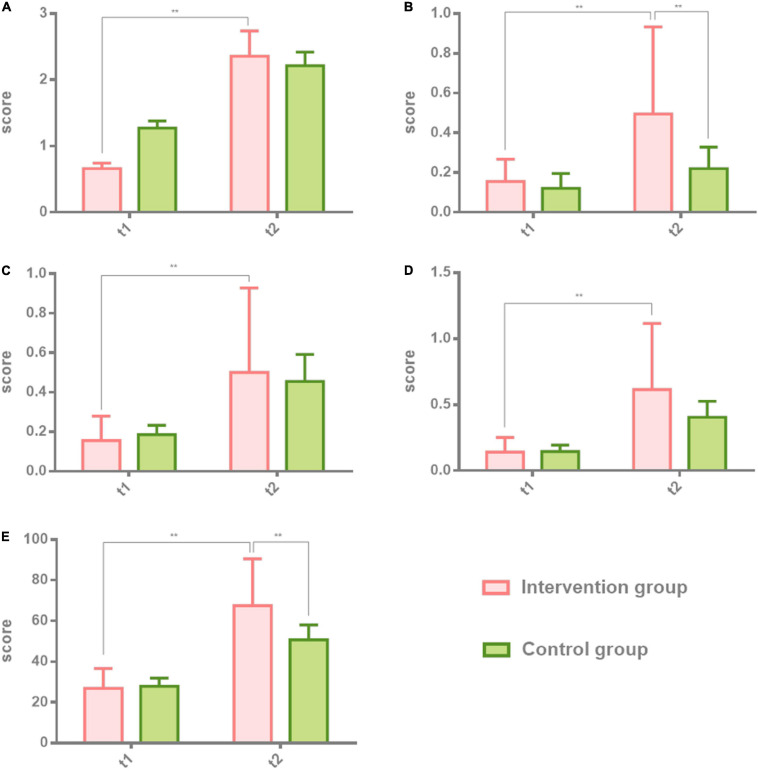
Comparison of naming and aphasia quotient (AQ) in patients with non-fluent aphasia with the intervention group and the control group. The intervention group: melodic intonation therapy group; the control group: speech therapy group. **(A–E)** Objective naming, spontaneous naming, sentence completing, reaction naming, and AQ. Data are expressed as mean ± SD (*n* = 20) and analyzed by repeated-measures analysis of variance. *p < 0.05, **p < 0.01. t_1_, baseline; t_2_, after 8 weeks.

The second part of the BDAE scale, naming and aphasia quotient (AQ), was tested in both groups before the first and after the last session. Two-way ANOVA was used to analyze the results of the intervention group and the control group at t1 and t2. Individual results were normalized by ruling out the difference of greater dispersion. The effect of the intervention group is higher than the control group. There were significant differences in the intervention group at t2 (8 weeks after) on objective naming (*t*_2_ = 2.36 ± 1.72, *t* = 0.4337, *p* = 0.0001), spontaneous naming (*t*_2_ = 0.50 ± 0.44, *t* = 3.698, *p* = 0.0001), sentence completing (*t*_2_ = 0.50 ± 0.43, *t* = 0.6079, *p* = 0.0001), reaction naming (*t*_2_ = 0.62 ± 0.50, *t* = 2.512, *p* = 0.0001), which belongs to naming. There were also significant differences in the intervention group at t2 (8 weeks after) on the AQ (*t*_2_ = 67.47 ± 22.99, *t* = 4.036, *p* = 0.0001) in comparison with the control group. Table 3 shows the results of the two groups. Figure 3 shows the comparison results of the two groups.

**TABLE 3:** HAMA and HAMD questionnaire results.

		Intervention		Control		*F*	*p*
		group (*n* = 20)		group (*n* = 20)			
		Mean	*SD*	Mean	*SD*		
HAMA	t_1_	12.15	3.16	12.75	2.47	0.1528	0.697
	t_2_	8.6	2.68	9.65	1.8	2.054	0.1559
HAMD	t_1_	16.15	2.52	16.45	2.25	3.028	0.0859
	t_2_	8.95	1.97	10.9	1.64	5.63	0.0202*

*p < 0.05 significant differences; (a) difference factor at the same time between groups. (b) difference effect of time factor in inter-group.

### Effectiveness of the Hamilton Anxiety Scale and Hamilton Depression Scale in Patients With Non-fluent Aphasia

**FIGURE 4:**
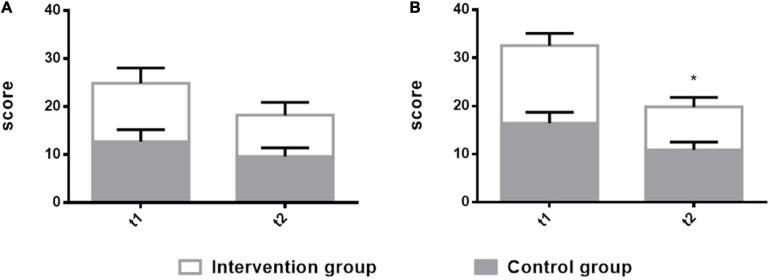
Comparison of the Hamilton Anxiety Scale (HAMA) and the Hamilton Depression Scale (HAMD) in patients with non-fluent aphasia between the two groups with melodic intonation therapy and speech therapy. The intervention group: melodic intonation therapy group; the control group: speech therapy group. **(A)** HAMA; **(B)** HAMD. Data are expressed as mean ± SD (*n* = 20) and analyzed by repeated-measures analysis of variance. *p < 0.05, **p < 0.01. t_1_, baseline; t_2_, after 8 weeks.

A main effect of time was found for the HAMA and the HAMD. The effect of the intervention group is higher than the control group. There was a significant difference at t2 (8 weeks after) on HAMD (*t*_2_ = 8.95 ± 1.97, *F* = 5.63, *p* = 0.0202) in the intervention group in comparison with the control group. There was no significant difference at t2 (8 weeks after) on HAMA (*t*_2_ = 8.6 ± 2.68, *F* = 2.054, *p* = 0.1559) in the intervention group in comparison with the control group. A significant difference was observed on HAMD at the t2 time point. Supplementary Table shows the results of the two groups. Figure 4 shows the comparison results of the two groups.

## Discussion

In this study, melodic intonation therapy and speech therapy were used as the therapeutic method in patients with aphasia. Compared with the previously reported method that used traditional speech therapy (Wan et al., 2014) as the therapeutic way to treat the patients with aphasia, this study was based on a long-time clinical work with aphasia patients that found that melodic intonation therapy has a positive effect in the clinic. The researcher of this study and the professional music therapists tried to use melodic-inducted speech output as the melodic auditory stimulation to intervene in the patients with aphasia and obtained unexpected results in comparison with the single-speech therapy group.

### Spontaneous Speech, Repetition, and Listening Comprehension

Speech therapy is a common training method for speech disorders in patients with aphasia (Albert et al., 1973). However, due to the limitations of the method and a single-training mode, it is actually difficult for patients to adhere to or results are slow. Due to the different mechanisms, MIT uses “singing” to guide and uses the method of chanting a melody formula language to guide patients with aphasia from “singing” to speak. It is generally believed that the right hemisphere is better at processing short melody information (Sihvonen et al., 2017). Therefore, for patients with stroke on the left, the right hemisphere plays a compensatory role in oral output when “singing.” MIT is, therefore, effective.

In BDAE, spontaneous speech, repetition, and listening comprehension are the test criteria for speech and listening ability. Spontaneous speech includes information, fluency, grammar, and paraphasia. Listening comprehension includes true or false questions, word recognition, and successive instructions. Repetition is a separate item, which includes 10 sentences of different lengths, includes words, short sentences, and long sentences; in the examination of this item, the patient only needs to imitate. The three sub-examinations of the BDAE scores were used to assess language comprehension and imitation in aphasia patients with visual and auditory cues. In the imitation of listening comprehension and speaking, MIT has a clearer immediate effect than speech therapy because it uses the mechanism of singing of music. The patient sings the target language while imitating the tone, which is more direct and effective than single vocabulary auditory stimulation. Therefore, in this study, the intervention group undertaking MIT had significant time cumulative effects and intergroup effects in terms of spontaneous language information, oral expression, imitation repetition, true or false judgment, hearing word recognition, and instruction execution than the control group undertaking speech therapy alone. In terms of improvement of spontaneous speech fluency, patients in the MIT group showed higher speech fluency after 8 weeks of cumulative treatment. However, compared with the control group, speech therapy also had similar effects on spontaneous speech fluency. It can be seen that the improvement of fluency is more due to the accumulation of effective treatment time. In other words, among the hospitalized non-fluent aphasia patients who received the same medical care, the music therapy–MIT group has better speech recovery effects in listening comprehension, repetition, and spontaneous speech than patients who received speech therapy and has a similar effect in terms of improving fluency.

### Naming and Aphasia Quotient

The BDAE’s naming test on abstract thinking is divided into four dimensions: objective naming, spontaneous naming, sentence completing, and reaction naming. Patients in the intervention group receiving MIT performed more prominently in spontaneous naming. After 8 weeks of intervention, they showed a significant cumulative effect of time. Compared with the control group, the intervention group showed a larger difference between groups. This is closely related to the repeated use of “singing” for intervention in the MIT treatment. Singing is a whole-brain activity; when the patient participates in singing, the cognitive information network related to the song will be activated (Merrett et al., 2014). Because the song contains more information, the listening experience of singing with an accompanying instrument is more complicated, not only for the language in the trained items but also for the language in other untrained items (Meulen et al., 2016). MIT can also activate more spontaneous naming responses. In terms of object naming, sentence completion, and reaction naming, the MIT group was also significantly different from the speech therapy group, but there was no obvious contrast effect in the cumulative effect after 8 weeks. Therefore, compared with MIT, speech therapy has consistent efficacy in non-spontaneous naming.

In conclusion, in the aphasia quotient performance of the two groups of patients through the BDAE test, compared with the control group using speech therapy, the overall score of the intervention group has an obvious improvement either in the time effect after 8 weeks or in the comparison between the two groups.

### Hamilton Anxiety Scale and Hamilton Depression Scale

In this study, the score of the HAMD of the intervention group was lower than that of the control group, and there is no significant difference in the score of HAMA, which means a more positive subjective experience of the music group and has a decrease feeling in depression. Patients in the intervention group reported decreasing feelings in expressive difficulty, and the falling ratings are accompanied by improved speech functionality. In the control group, ratings also declined, but it did not show significant differences compared with the intervention group. In the HAMD measurements, both of the groups reported a decrease in depressive symptoms, but patients’ score of the intervention group was lower than that of the control group. Each patient in the intervention group underwent an individual music therapy session that promoted interaction and positive experience. Singing interventions may reduce the depressive stress of the patients. Through familiar songs, singing, and accompaniment with music therapists, patients’ sense of satisfied and happy feelings will enhance during a singing session. Therefore, music therapy may have a positive effect on aphasia patients. Having an enjoyable musical training course not only motivates them to increase participation but also brings important emotional experience. The family and guardian of aphasia patients from the intervention group reported that the patients felt reconnected with life, either by singing more, or by exploring and listening to familiar songs, and had a greater sense of participation in music activities in life, as well as the link between music, health, and quality of life. This training method, based on music and singing, has a higher participation rate, is easier, simpler, and more effective for patients to comply.

### The Core of Melodic Intonation Therapy Intervention

Melodic guidance at MIT can be divided into two parts: in the first part, melody guides the language, the second, the musical language stimulation. In the process of melody in guiding the target language, the pitch of the melody comes from the natural pronunciation of Mandarin Chinese. For example, the three-tone “you” in Mandarin can be imitated by the interval of “sol–dol” (G-C). The patient began to sing and slowly generalized into speaking. These fixed-pitch formulaic melody languages range from 2 to 5 short sentences to 7–10 long sentences (Supplementary Materials), that is, to create lyrics of daily life language with a fixed melody, teach patients to sing, and then slowly get out of the melody, and the pitch becomes speaking. This is an entirely different approach from speech therapy intervention. According to the concept of “*sprechsang*,” first proposed by Sparks scholars in 1974 (Albert et al., 1973), the melody in MIT is between “singing” and “speaking.” In this study, the core intervention technology of MIT complies with the core principle of “*sprechsang*,” but the innovation lies in its application in Mandarin Chinese. The formation of pitch melody completely simulates the laws of Chinese phonetics.

In the second part, after MIT, if the patient can imitate the pitch but cannot imitate the Chinese character sound, they will use MUSTIM to sing familiar songs to guide the words that cannot be expressed verbally. The choice of the song is not blind. When the music therapist chooses the song, the lyrics will include the vocabulary of the target language. For example, when the patient is guided to say “drink water,” and the patient cannot complete it, the therapist will lead the patient to sing a song with the sound of “he” and “shui,” such as “Wanquan River Clear and Clear” (with lyrics “River water”-“drink water”) or “Love the country and the beauty more” (including the lyrics “drink the same water”). After the familiar cognitive melody is guided, the patient is guided back to the model singing of the formulaic melody, so that the patient can imitate and say it. This is why aphasia patients in the MIT group performed particularly prominently in the repetition items.

When the patient sings each short melody or song, it is accompanied by a guitar or piano and other harmonic instruments. Music therapists use musical instruments with human voices to guide patients to chant, which enriches patients’ auditory experience in auditory input. Due to the interaction of the left and right hemisphere networks when the brain processes language information, when the auditory center receives multiple stimulations, they jointly activate the output of emotion, memory, and spoken language. Therefore, in this study, the MIT group in the BDAE score has improved listening comprehension, repetition, spontaneous speech, and naming.

### Limitations

One limitation was the limited sample, as previously detailed. Two participants dropped out of the study, which may have caused the variance in group allocation. If a blank, the control group was added to observe self-healing, and the comparison might have been more accurate. This study only recruited 40 patients. If larger-sized studies are conducted in the future, the therapeutic outcomes could be more precisely observed. Besides, the participants with three different types of non-fluent aphasia are included into the trial. Although they belong to non-fluent aphasia, they also belong to different subtypes. If more samples can be included in future studies and different subtypes of aphasia are classified and compared, the effect comparison of the two methods will be clearer.

### Implications for Clinical Practice

In previous reports in the literature, clinicians usually recommend speech therapy to train the patients with aphasia but neglected that the function of song singing played an important role in speech output. Although MIT has been proposed and used in the 1970s, it is often used by speech therapists. Given that professionals with a musical background, that is, music therapists, will have a more professional understanding of music or songs, and the operability of the musical instrument, the MIT performed by the music therapist will provide multiple auditory stimulation to the patients to activate more potential brain networks and better restore language ability. Through this study, we confirmed the positive effect of the MIT performed by a music therapist in 20 aphasia patients. All the participants in the intervention group were more active in every aspect of AQ than the control group, which provided a more effective way for speech recovery of aphasic patients. Music therapists with professional backgrounds provide multiple auditory stimuli with instrumental accompaniment and fixed-pitch melody formulaic language during the treatment process, which are all necessary conditions for the implementation of MIT.

## Conclusion

The MIT performed by music therapists has a more obvious effect on improving the language function of patients with non-fluency aphasia. Therefore, it is recommended that clinicians and professional music therapists work together to make the clinical treatment effect more remarkable.

## Data Availability Statement

The original contributions presented in the study are included in the article/Supplementary Material, further inquiries can be directed to the corresponding author/s.

## Ethics Statement

This study was approved by the Ethics Committee of China Rehabilitation Research Center (Approval No. 2020-013-1 in April 1, 2020), and was registered with the Chinese Clinical Trial Registry (Registration number: Clinical Trials ChiCTR2000037871) on September 3th, 2020. The patients/participants provided their written informed consent to participate in this study.

## Author Contributions

W-JT and M-YL supported music therapy—MIT treatment in the intervention group. W-YY supported the assessment. W-ZW was the principal investigator of this project. X-LW, Y-QY, and L-XL supported the patients’ allocation. J-JL was the corresponding author. All authors contributed to the article and approved the submitted version.

## Conflict of Interest

The authors declare that the research was conducted in the absence of any commercial or financial relationships that could be construed as a potential conflict of interest.

## Publisher’s Note

All claims expressed in this article are solely those of the authors and do not necessarily represent those of their affiliated organizations, or those of the publisher, the editors and the reviewers. Any product that may be evaluated in this article, or claim that may be made by its manufacturer, is not guaranteed or endorsed by the publisher.

## Supplementary Material

Supplementary File 1 Aphasia Participants’ characteristics.

Supplementary File 2 Melodic intonation therapy musical pieces.

Supplementary File 3 Hospital Ethics Documentation.
