# Supplementary figures and images for: Effectiveness of Melodic Intonation Therapy in Chinese Mandarin on Non-fluent Aphasia in Patients After Stroke: A Randomized Control Trial
Source: Front Neurosci. 2021 Jul 23;15:648724. doi: 10.3389/fnins.2021.648724 (PMC8344357; doi:10.3389/fnins.2021.648724)

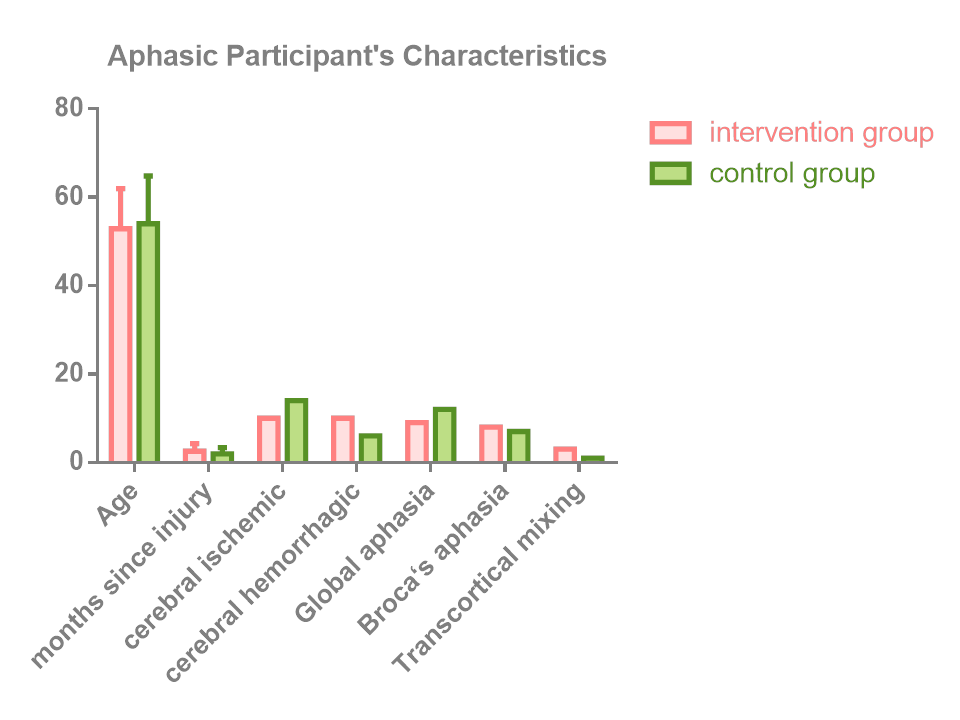

Supplement: Supplementary File 1 — Aphasia Participants’ characteristics. [file Image_1.TIF]

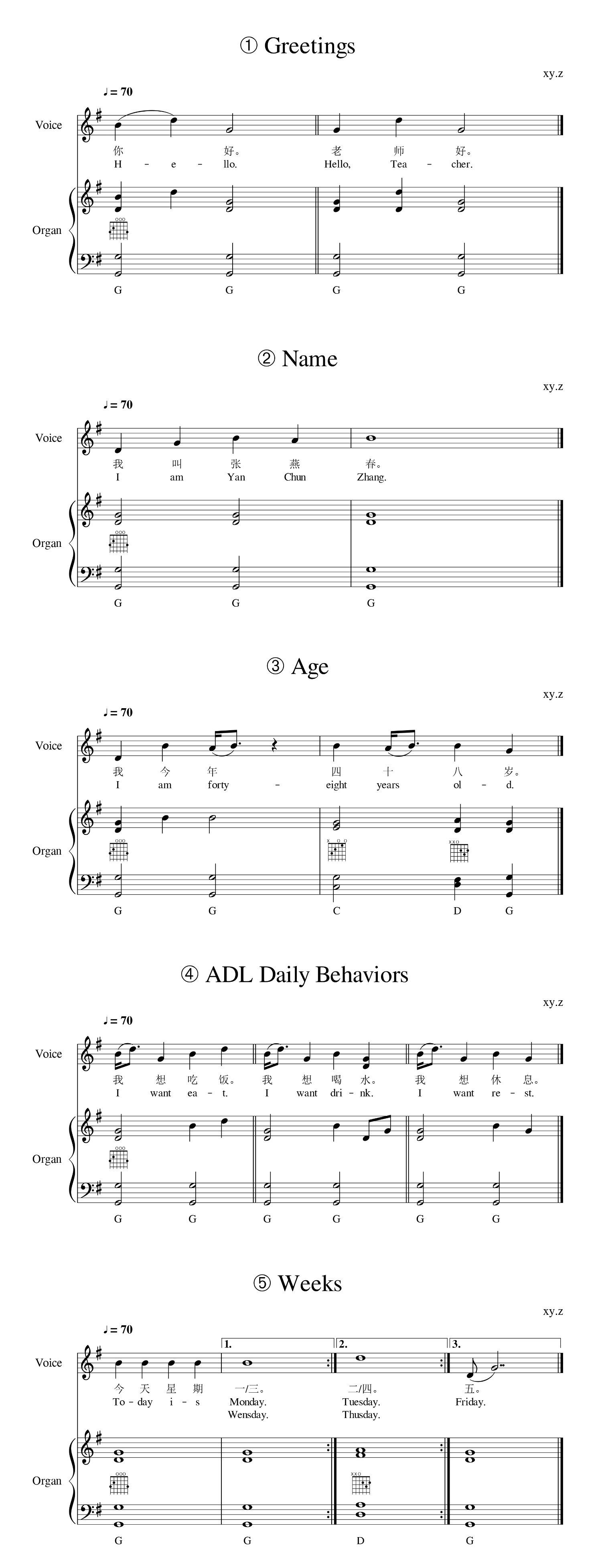

Supplement: Supplementary File 2 — Melodic intonation therapy musical pieces. [file Image_2.TIF]

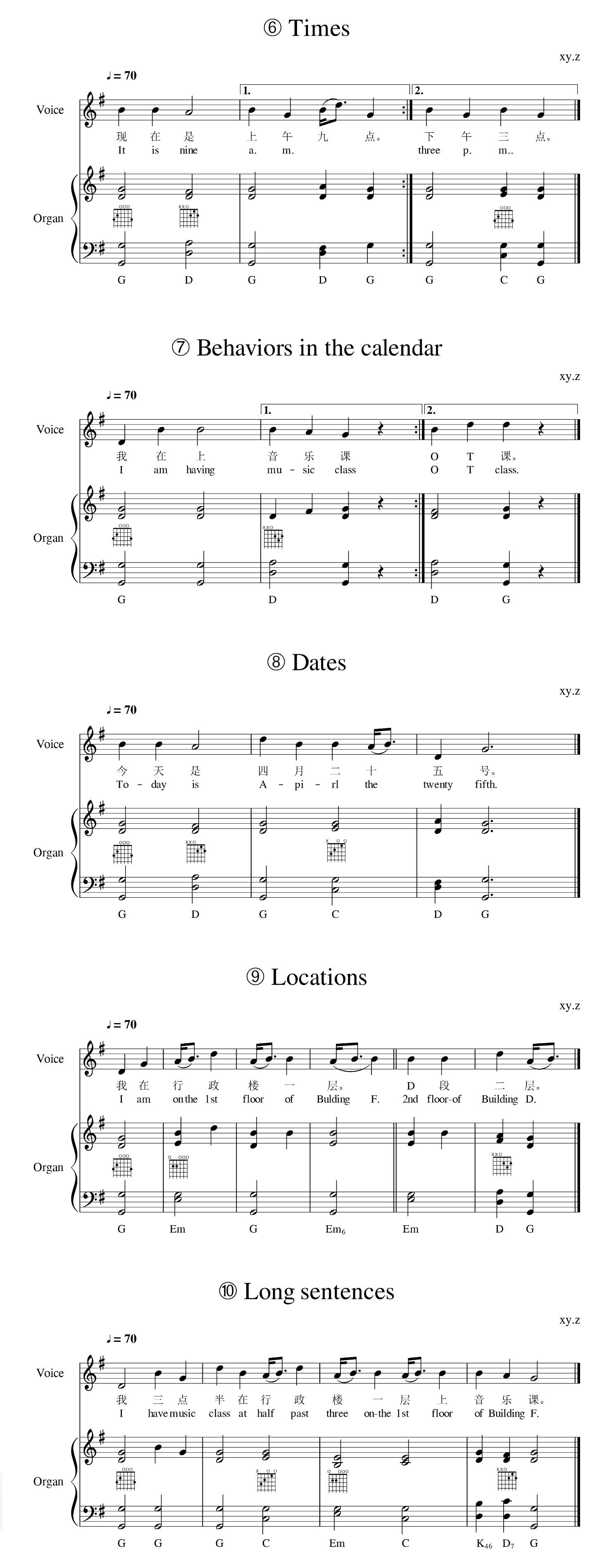

Supplement: Supplementary File 3 — Hospital Ethics Documentation. [file Image_3.TIF]
